# The Development of Nasal Polyp Disease Involves Early Nasal Mucosal Inflammation and Remodelling

**DOI:** 10.1371/journal.pone.0082373

**Published:** 2013-12-10

**Authors:** Juan Meng, Peng Zhou, Yafeng Liu, Feng Liu, Xuelian Yi, Shixi Liu, Gabriele Holtappels, Claus Bachert, Nan Zhang

**Affiliations:** 1 Department of Oto-Rhino-Laryngology, West China Hospital, West China Medical School, Sichuan University, Sichuan, China; 2 Upper Airway Research Laboratory, Department of Otorhinolaryngology, Ghent University Hospital, Ghent, Belgium; 3 Division of ENT Diseases, Clintec, Karolinska Institutet, Stockholm, Sweden; Hospital of the University of Pennsylvania, United States of America

## Abstract

Chronic rhinosinusitis with nasal polyps (CRSwNP) is characterized by both a chronic inflammation and tissue remodelling; as indicated by extracellular matrix protein deposition, basement membrane thickening, goblet cell hyperplasia and subepithelial edema, with reduced vessels and glands. Although remodelling is generally considered to be consequence of persistent inflammation, the chronological order and relationship between inflammation and remodelling in polyp development is still not clear. The aim of our study was therefore to investigate the pathological features prevalent in the development of nasal polyps and to elucidate the chronological order and relationship between inflammation and remodelling, by comparing specific markers of inflammation and remodelling in early stage nasal polyps confined to the middle turbinate (refer to as middle turbinate CRSwNP) obtained from 5 CRSwNP patients with bilateral polyposis, mature ethmoidal polyps from 6 CRSwNP patients, and normal nasal mucosal tissue from 6 control subjects. Middle turbinate CRSwNP demonstrated significantly more severe epithelial loss compared to mature ethmoidal polyps and normal nasal mucosa. The epithelial cell junction molecules E-cadherin, ZO-1 and occludin were also expressed in significantly lower amounts in mature ethmoidal polyps compared to healthy mucosa. Middle turbinate CRSwNP were further characterized by significantly increased numbers of subepithelial eosinophils and M2 type macrophages, with a distinct lack of collagen and deposition of fibronectin in polyp part. In contrast, the turbinate area of the middle turbinate CRSwNP was characterized by an increase in TGF-β activated myofibroblasts expressing α-SMA and vimentin, an increase in the number of pSmad2 positive cells, as well as increased deposition of collagen. These findings suggest a complex network of processes in the formation of CRSwNP; including gross epithelial damage and repair reactions, eosinophil and macrophage cell infiltration, and tissue remodelling. Furthermore, remodelling appears to occur in parallel, rather than subsequent to inflammation.

## Introduction

Nasal polyposis is a common inflammatory disease of the nasal and paranasal mucosa, affecting 1-4% population [1]. In Caucasians, chronic rhinosinusitis with nasal polyps (CRSwNP) is typically associated with a Th2 skewed eosinophilic inflammation with high IL-5 and ECP concentration in the mucosal tissue [2,3]. Furthermore, the Th2 signature is accompanied by a reduced transforming growth factor-beta (TGF-β) signalling, which can play a crucial role in both the suppression of airway inflammation and remodelling [4,5]. Thus, CRSwNP is characterized by both a chronic inflammation and changes in ECM protein deposition and tissue structuring; observed as basement membrane thickening, goblet cell hyperplasia and subepithelial edema, with reduced vessels and glands.

Remodelling has long been considered to be a secondary phenomenon and developing late in the disease process as a consequence of persistent inflammation. However, findings from our studies suggest that there may not be a direct link between inflammation and remodelling in upper airway disease. A comparison of the inflammatory patterns in the upper airways of Caucasian and Chinese CRSwNP patients demonstrated that there were clear differences in the inflammatory patterns in these ethnicity groups, although the remodelling patterns appeared to be similar [6]. In particular, the Chinese patients were biased towards neutrophilic inflammation and showed a significant increase in Th1/Th17 cell patterns, while the Caucasian patients showed significantly increased Th2 cytokine profile [7]. In contrast, the remodelling patterns in Chinese CRSwNP patients were similar to Caucasian CRSwNP patients; with lower concentrations of TGF-β1, TIMP-1, TIMP-4, and reduced deposition of collagen [5]. It has been demonstrated that fibroblasts and myofibroblasts play an important role in the remodelling process associated with chronic airway inflammation, including CRSwNP [8,9]. Furthermore, TGF-β1 is able to induce proliferation and differentiation of fibroblasts into myofibroblasts [10,11], which have tissue contractile properties and a high capacity for ECM protein secretion. A study by Wang and colleagues [11] investigating the histopathology of nasal polyps in CRSwNP patients has demonstrated that although myofibroblasts were more abundant in the pedicle region of the nasal polyps than in the central and tip areas of CRSwNP, these were localized with TGF-β in all regions. These results suggest that the local development and involvement of myofibroblasts in nasal polyps may be mediated by TGF-β. Similarly, findings from our study [6] suggest that the release of collagen and the balance between metalloproteinases and their inhibitors in nasal polyps may also be mediated by TGF-β1.In view of these findings we aimed to investigate the pathological features prevalent in the outgrowth of nasal polyps and to elucidate the chronological order and relationship between inflammation and remodelling; by comparing specific markers of inflammation and remodelling in early stage nasal polyps confined to the middle turbinate in CRSwNP(refer to as middle turbinate CRSwNP) patients with bilateral polyposis, mature ethmoidal polyps in CRSwNP patients, and normal nasal mucosal from control subjects. 

## Materials and Methods

### Subjects

A total of 17 subjects (6 CRSwNP patients with mature ethmoidal polyps, 5 patients with CRSwNP and early polyp formation on the middle turbinates, and 6 control subjects who were undergoing rhinoseptoplasty because of anatomical variations and were not suffering from any sinus disease) were recruited into the study at the department of Oto-Rhino-Laryngology of the University Hospital of Ghent, Belgium. The diagnosis of chronic rhinosinusitis with polyps (CRSwNP) was based on the history, clinical examination, nasal endoscopy and computed tomography according to the current EP3OS guidelines [12]. Atopy was assessed by skin prick test to common inhalant allergens and all patients were asked to stop taking antibiotics, oral and topical corticoid steroids for at least 1 month before surgery.

**Figure 1:**
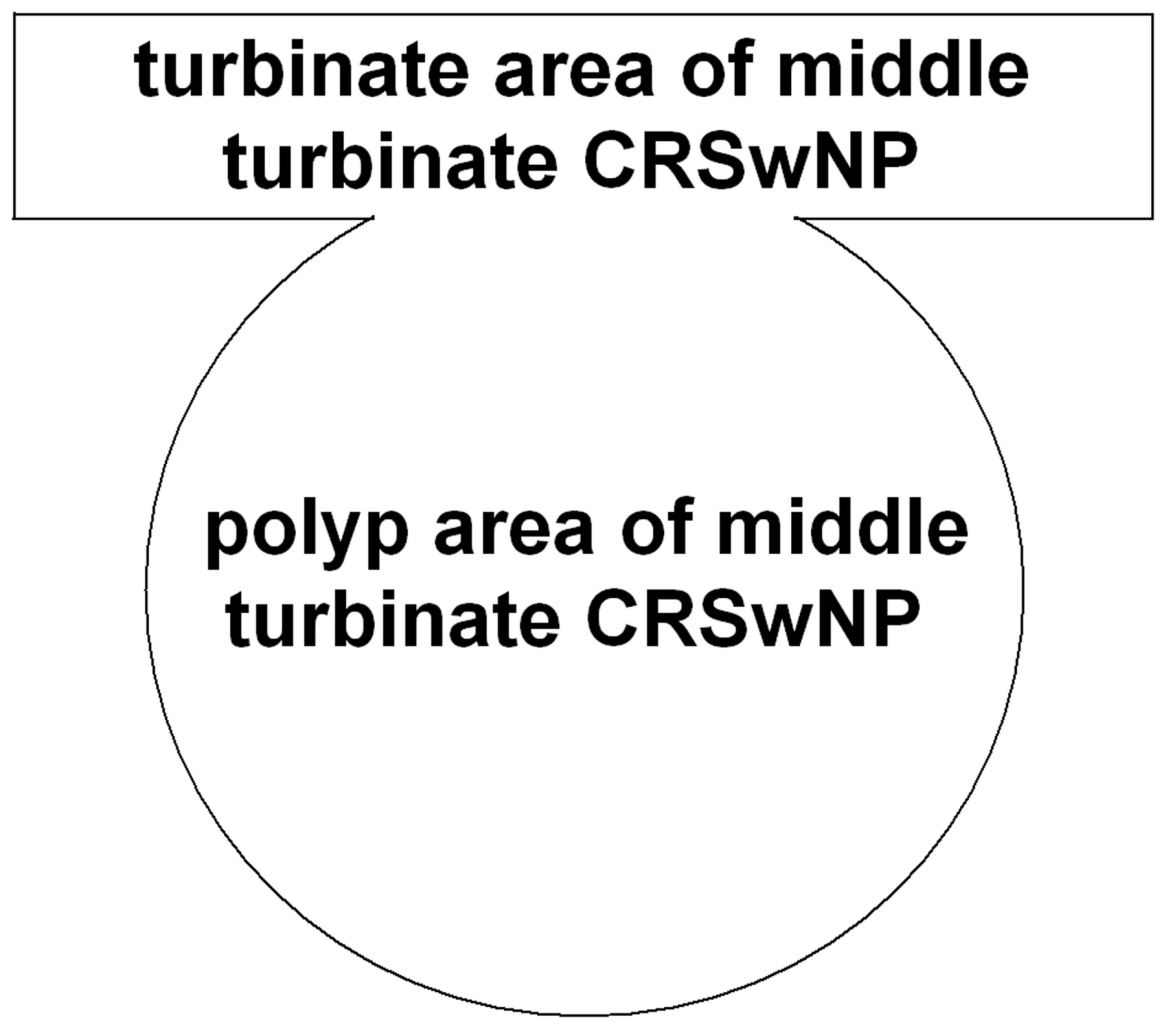
Diagram of middle turbinate polyps in patients with CRSwNP. As the diagram shows, for the samples of middle turbinate CRSwNP, special attention was paid to the collection of the whole polyp with the stalk and parts of the surrounding mucosa. This allowed us to discriminate into polyp area and turbinate area for analysis.

As controls, samples were obtained from the inferior turbinate during septal surgery. For the middle turbinate CRSwNP specimens, samples were taken from patients with bilateral CRSwNP and small nasal polyp formation on the medial aspect of the middle turbinate. These polyps were considered to occur at a rather late stage in CRSwNP, and thus of younger age (early stage) than the mature ethmoidal specimens from the selected CRSwNP patients. Special attention was paid to collecting the whole polyp with the stalk and parts of the surrounding mucosa (Figure 1). 

The study was approved by the ethical committee of the Ghent University Hospital and performed at Ghent University Hospital, Ghent, Belgium. All patients gave written informed consent before participation.

### Immunohistochemistry

Tissue was fixed in formalin (Fluka, Sigma-Aldrich, Bornem, Belgium) and embedded in paraffin, prior to being cut at a thickness of 4mm and being air-dried for 24 hours at 37°C on microscope slides. The dried tissue sections were deparaffinised by heating in citrate buffer (pH= 6), and endogenous peroxidase activity was blocked by incubation with hydrogen peroxidase (Dako Envision + kit) for 5 minutes at room temperature. The sections were then washed with TBS for 10 minutes and incubated overnight at 4°C in the presence of one of the primary mouse or rabbit antihuman monoclonal antibodies for detection of a marker of interest as shown in Table 1. Negative controls for each marker of interest were prepared by incubating tissue sections overnight at 4°C in the presence of rabbit immunoglobulin fraction (Dako, Glostrup Denmark) or mouse IgG2a (Dako, Glostrup Denmark) antibodies. Following reaction in the presence of primary antibodies, the tissue sections were washed for 10 minutes in TBS and incubated for 20 min at room temperature in the presence of biotinylated anti mouse or anti rabbit (Dako LSAB+HRP-Kit, Dako North America, Capinteria, CA, USA) antibodies. At the end of incubation, the sections were washed again in TBS for 10 minutes, and then incubated for a further 20 min at room temperature in the presence of streptavidin –peroxidase (Dako LSAB+HRP-Kit). The streptavidin –peroxidase conjugated samples were washed in TBS for 10 minutes to remove any excess streptavidin –peroxidase, and then incubated with DAB+ substrate-chromogen (Dako North America, Capinteria, CA, USA) for 10 minutes to develop the colour to indicate the presence of the marker of interest. The sections stained for the presence of fibronectin (Fn) were incubated with DAB+ substrate-chromogen for 5 minutes, and all sections were counterstained with Haematoxyline (Sigma-Aldrich, Bornem, Belgium) for 2 minutes, prior to being washed under running tap water for 5 min, and mounted in Aquatex (Merck KGaA) for microscopic examination. 

**Table 1:** Information of the primary antibodies for IHC.

Antibody	Titer	Source	Catalog number	Function
E-cadherin	1:100	Invitrogen, Carlsbad, CA, USA	13-1700	Adherence junction of epithelial cell
ZO-1	1:200	Invitrogen,Carlsbad, CA, USA	61-7300	Tight junction of epithelia cell
Occluding	1:200	Invitrogen, Carlsbad, CA, USA	71-1500	Tight junction of epithelia cell
CD68	1:1	Dako, Glostrup Denmark	N1577	Marker for macrophage
mannose receptor (MMR)	1:1000	Abcam, Cambridge, UK	ab117644	Marker for M2 macrophage
α-smooth muscle actin(α-SMA)	1:100	Dako,Glostrup Denmark	M0851	Marker for myofibroblast
vimentin	1:200	Dako, Glostrup Denmark	M0725	Marker for myofibroblast
pSmad2	1:1000	Cell Signaling Technology, Beverly MA	Ser 465/467	Downstream marker for active TGF-βsignaling
Fibronectin(Fn)	1:2000	Dako, Glostrup Denmark	A0245	one of components of the ECM

### Picrosirius red staining for collagen

Collagen was assessed by picrosirius red staining as previously described [5] . After deparaffinization, tissue sections were stained with picrosirius red for one hour, and then washed twice in acidified water and dehydrated in three changes of 100% ethanol. After clearing in xylene, the sections were mounted in Aquatex (Merck KGaA). 

### Image analysis

#### Epithelial Loss

Analysis of epithelial loss was performed in Haematoxylin-Eosin (HE) stained samples, viewed at x100 magnification microscope. Images of the entire epithelium of a specimen were recorded on a digital camera (Olympus C-5050, Aartselaar, Belgium) without overlapping zones and analyzed using ImageJ software (Rasband WS. ImageJ, U.S. National Institutes of Health, Bethesda, Maryland, USA, imagej.nih.gov/ij/, 1997-2012).The length of missing epithelium and the total length of the basement membrane were measured, and epithelial loss was expressed as the percentage of the missing epithelium length over total basement membrane length. The epithelium was considered incomplete when the basement membrane was completely denuded or when it was covered by only a single layer of basal cells with no intact ciliated cells or goblet cells [13].

#### E-cadherin, ZO-1 and occludin

10 random images(x400 magnification) from each sample were recorded on a digital camera (Olympus C-5050, Aartselaar, Belgium) without overlapping zones, and then assessed by two independent observers blinded to the tissue staining protocol (for all the staining below, this step was the same). As most all of the epithelium was missing in the polyp area of the middle turbinate samples, it was not possible to include these samples in the assessment, and a semi-quantitative analysis was therefore performed on the intact areas of epithelium in other samples, using an arbitrary 5-point visual staining scale as follows: 0 absent; 1 weak; 2 moderate; 3 intense; and 4 very intense staining [14].

#### Counts for eosinophil cell, α-SMA, vimentin, CD68 and macrophage mannose receptor (MMR) positive cells

Eosinophils were counted in HE stained sections. For α-SMA and vimentin positive cells, only scattered positive cells detected in the lamina propria were counted, while immune-labelled cells arranged in groups around vessels or glands were not taken into account [11]. 

10 random images(x400 magnification) from each sample were recorded on a digital camera (Olympus C-5050, Aartselaar, Belgium) without overlapping zones, and then assessed by two independent observers blinded to the tissue staining protocol. The results were expressed as the number of cells per field at x400 final magnification.

#### pSmad2

10 random images(x400 magnification) from each sample were recorded on a digital camera (Olympus C-5050, Aartselaar, Belgium) without overlapping zones, and then assessed by two independent observers blinded to the tissue staining protocol, using ImageJ software as we have described previously [4].Briefly, original images were converted to an 8-bit grayscale and then using the threshold function, pSmad2 positive areas were converted to saturated black areas while all other areas were rendered white to result in a binary image. The threshold setting was manually validated by comparing the binary image to the original image. Positive nuclei were scaled using the function ‘‘analyze particles’’ (with a minimum size of 50 to a maximum of 500 pixels), and a report was generated for each image representing the count of pSmad2 positive cells.

#### Fibronectin (Fn)

 10 random images(x400 magnification) from each sample were recorded on a digital camera (Olympus C-5050, Aartselaar, Belgium) without overlapping zones, and then assessed by two independent observers blinded to the tissue staining protocol. Fibronectin staining in ECM was assessed semi-quantitatively on a 4-point staining intensity score as follows: 0, negative; 1, mildly positive; 2, intermediately positive; 3, strongly positive [15].

#### Picrosirius red

The sections were analysed with a microscope equipped with filters to provide circularly polarized illumination. The lower filter was placed above the microscope’s field iris diaphragm ring, while the upper filter was placed below the linear polarizer aligned such that its transmission axis was at 45. Collagen content was quantified under polarized light microscopy. 10 random images from each sample were recorded under bright-field at a final magnification of 400x without overlapping then analysed using ImageJ software. After subtraction of the background, original images were converted to an 8-bit grayscale. The total collagen amount was calculated for each section expressed as percentage of the total area [4]. The types of collagen fibres were determined under polarized light microscopy.

### Isolation and stimulation of fibroblasts from nasal tissue

Nasal tissues harvested from control subjects and CRSwNP patients with mature ethmoidal polyps were rinsed and suspended in sterile RPMI 1640 tissue culture medium (Sigma Aldrich, Bornem, Belgium); supplemented with 2mM L-glutamine (Invitrogen, Merelbeke, Belgium), 50IU/ml penicillin and 50mg/ml streptomycin (Invitrogen, Merelbeke, Belgium) and 0.1% bovine serum albumin ( Sigma Aldrich, Flanders, NJ, USA). The tissues were cut into small pieces and then incubated (at 37°C in a 5% CO2 in air atmosphere) in PBS containing 0.25% trypsinase for 45 min. Following this enzyme treatment, the tissues were gently passed through a cell mesh filter and washed twice by centrifugation at 10g and suspension in PBS. The final washed cells were suspended in RPMI 1640 supplemented with 2mM L-glutamine, 50IU/ml penicillin and 50mg/ml streptomycin, 10% foetal calf serum, and plated in a 25mm^2^ tissue culture flask (BD FalconTM, BD Biosciences) for 4 days. At the end of this incubation period, the non-adherent cells were removed by change of the culture medium and the attached cells cultured further for 2 weeks, by which time the cells had established as a confluent monolayer of fibroblast-like cells. The cells were harvested and cultured further for 3-5 passages, before being used in further experiments. During culture and in between the passages, the medium was changed every 3 days.

At the end of culture, the cells were harvested, seeded into 6-well plates at a concentration of 1×105Cells /well, and stimulated by incubation in the presence of 5ng/ml TGF-β1 (Sigma Aldrich, Flanders, NJ, USA), for 48h at 37°C in a 5% CO2 in air atmosphere. For each sample in each experiment, a negative control was also employed using cells incubated with only culture medium. At the end of culture the cell cultures were centrifuged at 4°C and the supernatants collected and stored at -20°C until use. One proportion of the stimulated cells was tested for the expression of α-SMA by flow cytometry and another proportion was collected for total RNA extraction and stored at -80°C.

### Enzymatic immunoassays

Concentrations of fibronectin in cell culture supernatants were measured by Elisa (Sigma Aldrich, Flanders, NJ, USA) according to the manufacturer’s instructions.

### Flow cytometry

The expression of α-SMA was detected by flow cytometry. The cells were adjusted to a concentration of 10^6^/ml and 100μL of supernatant was collected. Cells were fixed and permeabilized using permeabilizing solution 2(BD Bioscience, San Diego, California, USA) and then stained with 10μL of anti-human α-SMA -FITC (Abcam) for 20 min at room temperature. Corresponding isotypes were used as control. The cells were washed twice and analysed by flow cytometry (FACS, BD Bioscience).

### Quantitative real-time polymerase chain reaction

Total RNA was isolated using RNAisoplus ((TaKaRa, Dalian, China), and cDNA was synthesized from 5μg of RNA using the SuperScript® III First Strand Synthesis Kit (Invitrogen) in accordance with the manufacturer’s instructions. Amplification reactions were performed with Platinum® Taq DNA Polymerase High Fidelity (Invitrogen) using specific primer sets (homo-fibronectin1-F GACCAATGCCAGGATTCAGAGAC, homo-fibronectin1-R GACAGGACCACTTGAGCTTGGATAG). The PCR protocol consisted of 1 cycle at 95°C for 2 min followed by 40 cycles each at 95°C for 10 seconds, 60°C for 30 seconds, and 72°C for 30 seconds. β-actin, hydroxymethyl-bilane synthase, and elongation factor 1 were used as internal control genes for normalization. The relative number of molecules for each gene was expressed in relative expression units quantified per 20 ng of cDNA sample and determined by the △△ct value method.

### Statistical analysis

Statistical analysis was performed using the SPSS 11.0 (SPSS,Inc, Chicago, IL, USA). Data are expressed as box-and-whisker plots presenting medians, ranges, and interquartile ranges. Baseline variables were analyzed using a one-way ANOVA test and the Fisher’s exact text. The Kruskal-Wallis test was used to assess the significance of intergroup variability using paired comparisons and the Mann-Whitney U 2-tailed test was used for between-group comparisons. P-values <0.05 were considered statistically significant. 

## Results

### Patient characteristics

The demographic and clinical characteristics of the subjects in the three study groups are shown in Table 2. All groups were comparable in terms of age and female/male ratio. SPT positivity was observed in both CRSwNP groups; with only some CRSwNP patients with mature ethmoidal polyps having asthma, and none having COPD or being aspirin intolerant.

**Table 2:** Patient characteristics.

	Control	Mature CRSwNP	Middle turbinate CRSwNP	1-way ANOVA (Fisher exact test)
No.of patients	6	6	5	
Age mean (range)	31.2 (20-43)	39.8 (26-56)	38.6 (22-59)	NS
F/M	2/4	2/4	¼	NS
Asthma in history(n/N)	0/6	2/6	0/5	NS
SPT positive(n/N)	0/6	3/6	2/5	NS

### Epithelial assessment

**Figure 2:**
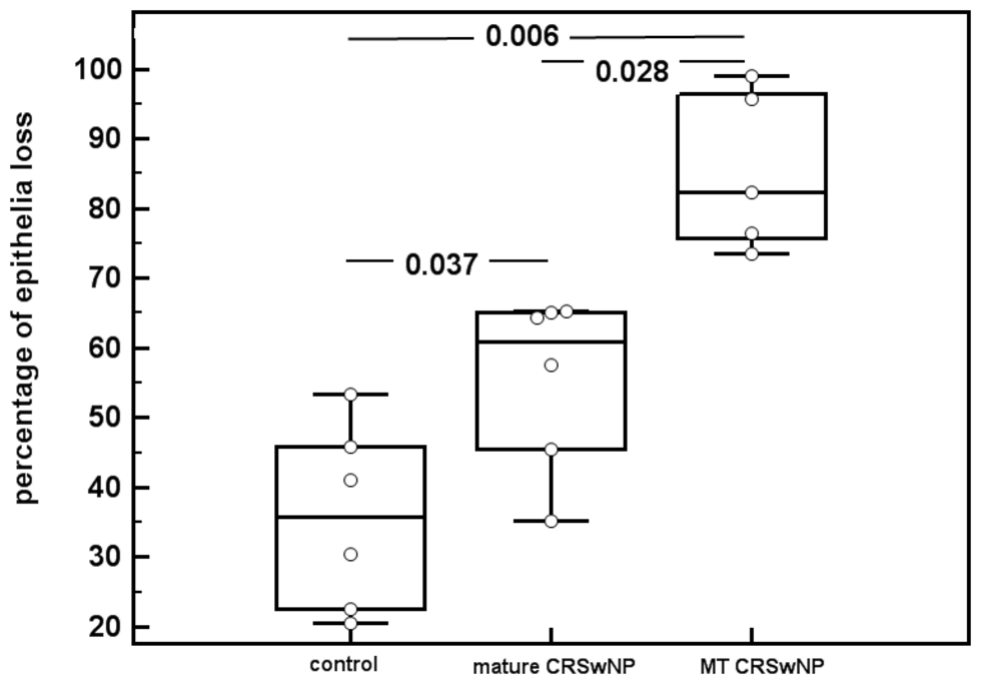
Epithelial loss (%) in control, mature CRSwNP and middle turbinate CRSwNP. MT, middle turbinate.

Assessment of the epithelial loss indicated that both the mature ethmoidal CRSwNP (median 60.93% , IQR 42.94-65.17%) and middle turbinate CRSwNP groups (median 82.30%, IQR 70.00-97.35%) demonstrated a significantly higher epithelial loss compared to the control group (median 35.83%, IQR 22.00-47.66%; *p*=0.037 and *p*=0.006, respectively). Furthermore, with the epithelial loss was significantly greater in the middle turbinate polyp compared to the mature ethmoidal polyp (*p*=0.028, Figure 2).

**Figure 3:**
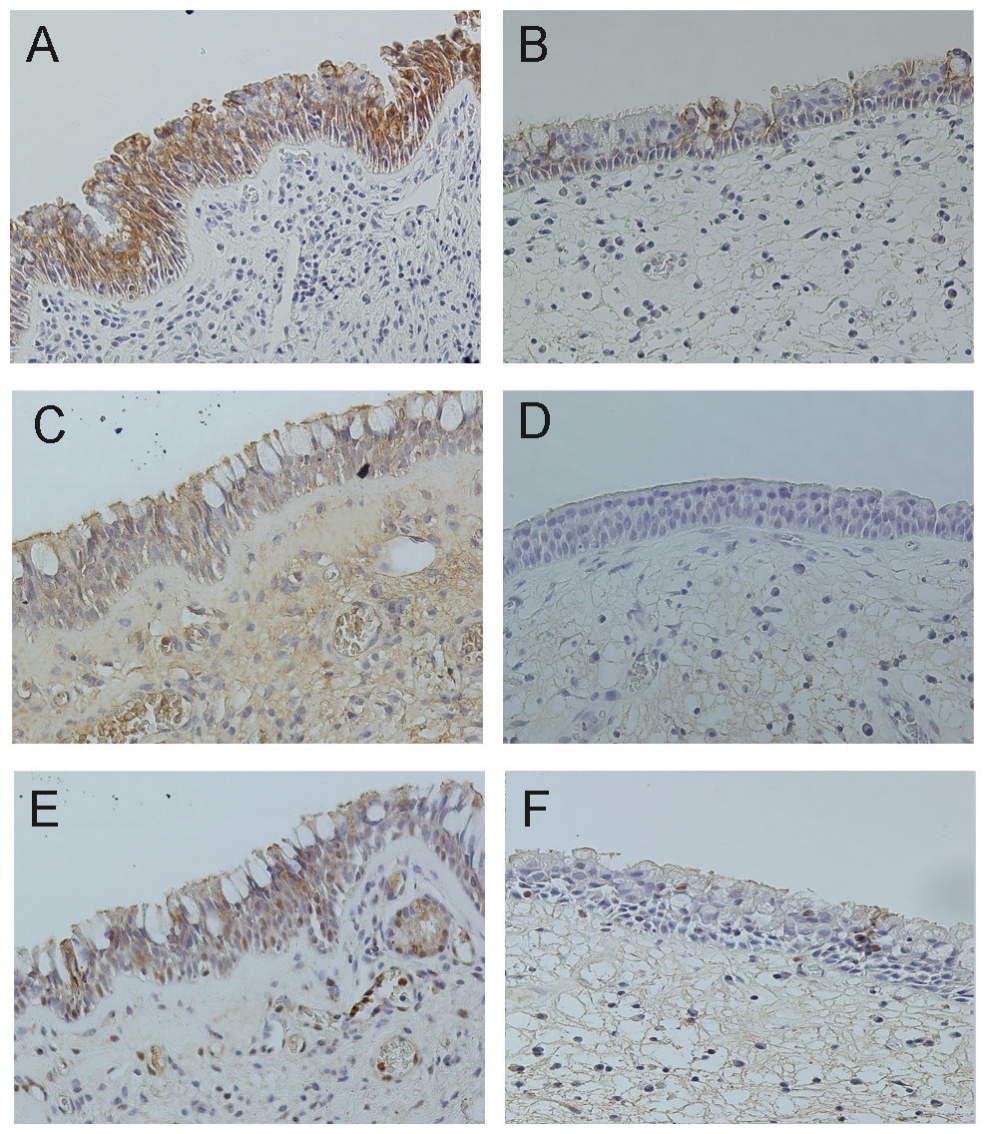
Immunostaining of E-cadherin, ZO-1 and occludin in control and mature CRSwNP. E-cadherin (A, B), ZO-1(C, D), occludin (E, F) for control (A, C, E) and mature CRSwNP (B, D, F).Original magnification 400x.

**Figure 4:**
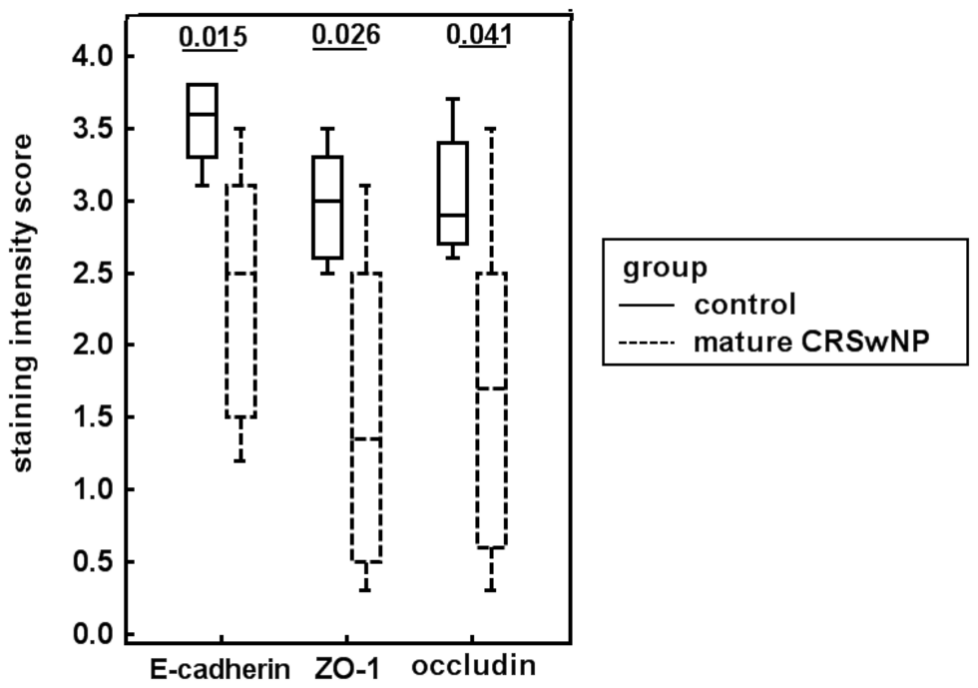
Quantification of the staining intensity for E-cadherin, ZO-1 and occludin in control and mature CRSwNP.

In normal nasal mucosa, E-cadherin was localized along the epithelial cells especially in the intermediate layer, sparing the basal cells (Figure 3A), whereas the distribution was segmental in the mature ethmoidal polyp (Figure 3B) and significantly lower in staining intensity compared with control mucosa (*p*=0.015, Figure 4). Similarly, assessment of the expression of ZO-1 (Figure 3C and 3D) and occludin (Figure 3E and 3F) demonstrated a significantly lower staining intensity for these proteins in the mature ethmoidal polyp tissue (Figure 3D, Figure 3F, respectively), compared to nasal mucosal tissue from controls (Figure 3C and 3E, respectively; *p*= 0.026 and *p*=0.041, respectively, Figure 4). In contrast, although epithelium in the turbinate area of middle turbinate CRSwNP appeared to be similar in nature to that in the control mucosa, almost all of the epithelium was missing in the polyp area of the middle turbinate CRSwNP . Thus it was not possible to evaluate or compare the intensity of the staining for any of these epithelial markers with that in either the mature ethmoidal polyps or the healthy nasal mucosa. 

### Assessment of cellular components

**Figure 5:**
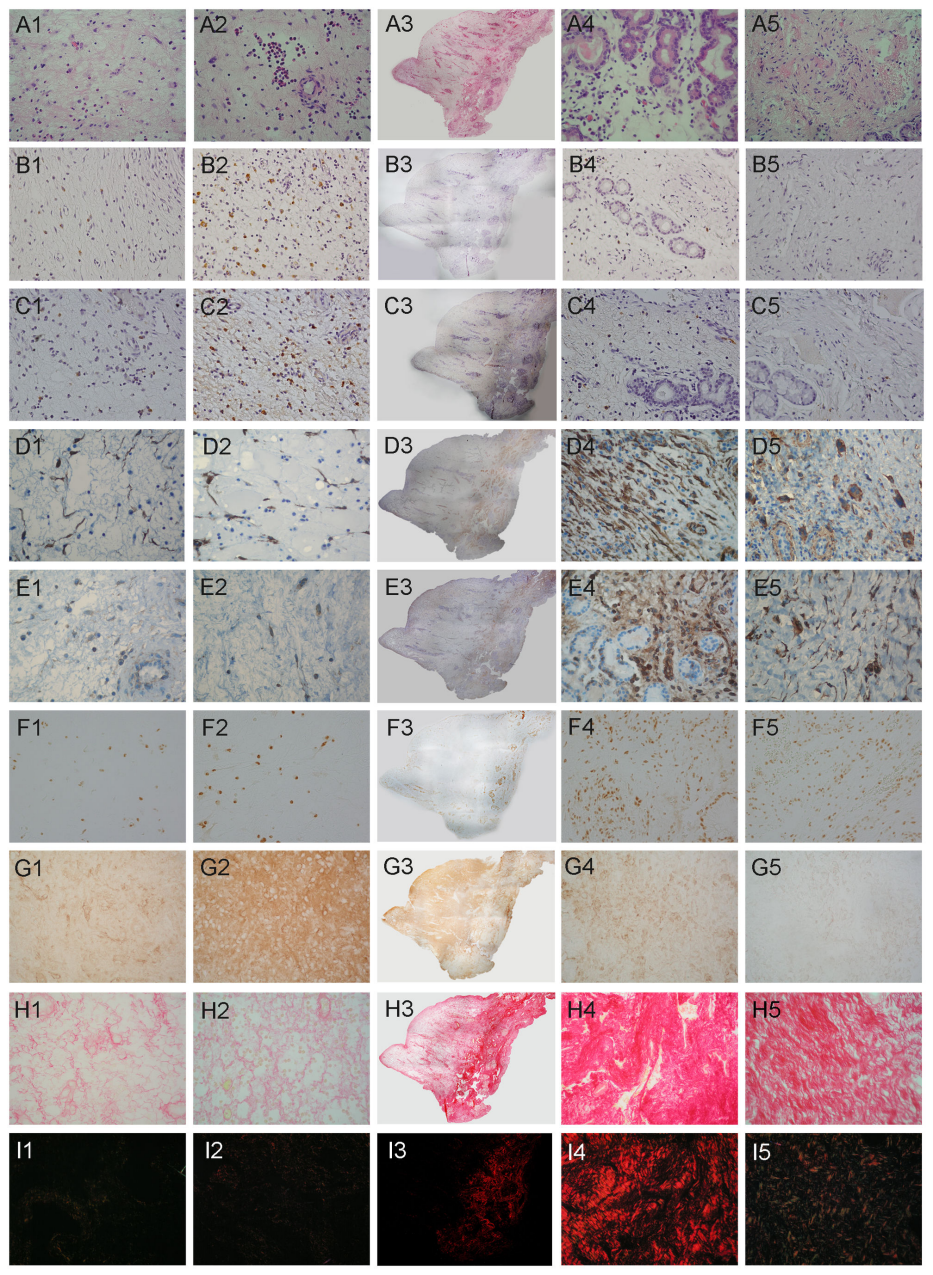
Quantification of celluar and extracelluar components. Eosinophils(A), CD68-(B), MMR-(C), α-SMA-(D), vimentin-(E), and pSmad2(F) positive cells, Fn (G) and collagen(H) in control interior turbinates (control IT), mature CRSwNP and middle turbinate CRSwNP, separated into turbinate and polyp areas.

Assessment of eosinophils showed that the number of eosinophils was significantly higher in the mature ethmoidal polyp CRSwNP group compared to the control group (*p*=0.006, Figure 5). In the early stage NP in the middle turbinate CRSwNP group, numerous eosinophils were mainly localized near the basement membrane of the polyp, but not within the middle turbinate area itself (*p*=0.009, Figure 5).

Similarly, the mature ethmoidal polyp CRSwNP group showed a significantly higher expression of CD68 and MMR-positive macrophages compared to the control group (*p*= 0.008 and *p*=0.006, respectively, Figure 5). A significantly greater accumulation of CD68 and MMR-positive cells was also noted in the turbinate part of middle turbinate CRSwNP group compared to the control group (*p*= 0.006 and *p*=0.006, respectively, Figure 5). Moreover these cells were additionally significantly higher in the polyp part compared to the turbinate part (*p*= 0.047 and *p*=0.028, respectively, Figure 5). Comparison with the mature CRSwNP further demonstrated that CD68 and MMR-positive cells were also significantly more pronounced in the polyp part of middle turbinate CRSwNP group than in the mature CRSwNP group (*p*= 0.006 and *p*=0.006, respectively, Figure 5).

Evaluation of the number of α-SMA positive cells, resembling myofibroblasts, indicated that these cells were significantly higher in the nasal mucosa in the control group than in mature CRSwNP group (*p*=0.004, Figure 5). In the middle turbinate CRSwNP, the distribution of α-SMA positive cell was limited to the turbinate area and was significantly greater than in controls (*p* =0.035, Figure 5). In contrast the distribution of these cells was scattered and sparse within the polyp part of the middle turbinate (*p*=0.009, Figure 5). 

Vimentin positive cells were found to be significantly higher in the turbinate part of the middle turbinate CRSwNP group compared to the mature CRSwNP group (*p*=0.017, Figure 5) and with a tendency, although not reaching significance, to be higher than normal control (*p*=0.054, Figure 5). 

The number of pSmad2 positive cells was significantly higher in control nasal mucosa compared with the mature ethmoidal polyp tissue (*p*=0.030, Figure 5), and in the turbinate part of middle turbinate CRSwNP compared to the polyp part of the middle turbinate CRSwNP (*p*=0.021, Figure 5). There was also not significant but a tendency to have more accumulation in turbinate part of middle turbinate CRSwNP compared to normal tissue (*p*=0.055, Figure 5).

### Assessment of extra cellular components

The mature CRSwNP demonstrated significantly greater levels of fibronectin than the control group (*p*=0.029, Figure 5). Similarly, the polyp part of the middle turbinate CRSwNP demonstrated significantly greater levels of fibronectin than the turbinate part (*p*=0.000, Figure 5). Moreover, the polyp part of middle turbinate CRSwNP also exhibited significantly greater levels of fibronectin than the mature CRSwNP (*p* =0.005, Figure 5).

**Figure 6:**
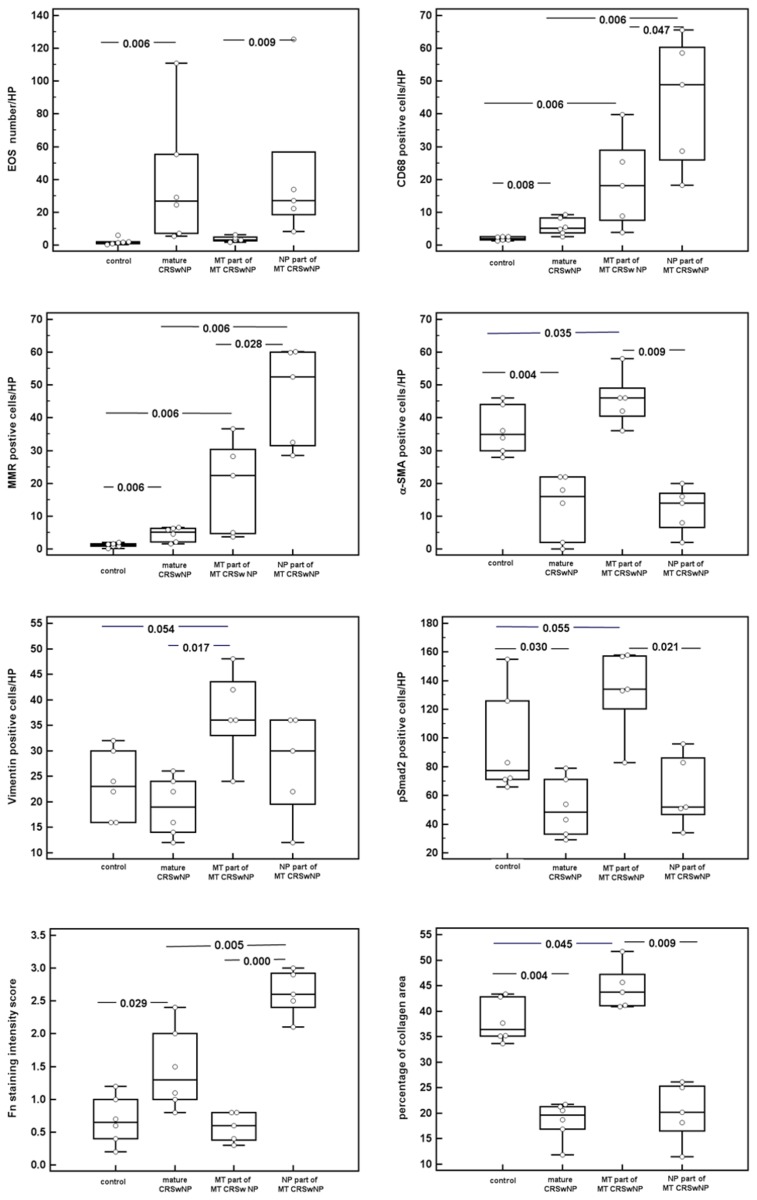
Immunostaining of celluar and extracelluar components. HE (A1-A5), CD68 (B1-B5),MMR (C1-C5), α-SMA (D1-D5), vimentin (E1-E5), pSmad2 (F1-F5), Fn (G1-G5) picrosirius red staining viewed in bright-field (H1-H5) and viewed under polarized light (I1-I5) in mature CRSwNP (A1-G1), polyp area of middle turbinate CRSwNP (A2-G2),turbinate area of middle turbinate CRSwNP (A4-G4) and control mucosa (A5-G5)(final magnification 400×). The overview of middle turbinate CRSwNP was shown in A3-G3 (composed by Photoshop software with pictures in 40×magnification).

Picrosirius red staining was performed to assess the collagen content within the ECM, which was evaluated by viewing the sections by bright-field microscopy, as shown in Figure 6, H1-H5. The total collagen expression in the ECM was found to be significantly higher in controls than mature CRSwNP (*p*=0.004, Figure 5), and also significantly higher in the turbinate area of middle turbinate CRSwNP than in the polyp part of the middle turbinate CRSwNP (*p*=0.009, Figure 5). Moreover, the turbinate part of middle turbinate CRSwNP was found to have significantly higher accumulation of collagen than the control group (*p*=0.045, Figure 5). Sections were additionally examined through crossed polar (Figure 6, I1-I5). Large collagen fibres light up in bright orange and thinner fibres show green. This birefringence, also called double refraction in highly specific for collagen. In this regard, orange collagen fibres which arraying in parallel, forming thick and dense fibre bundles, were present in the turbinate part of middle turbinate CRSwNP. In contrast, greenish luminescence was observed in control tissue, indicating the presence of thinner firers which forming a rather loose reticular structure.

### TGF-β1 stimulation of control and CRSwNP fibroblasts

**Figure 7:**
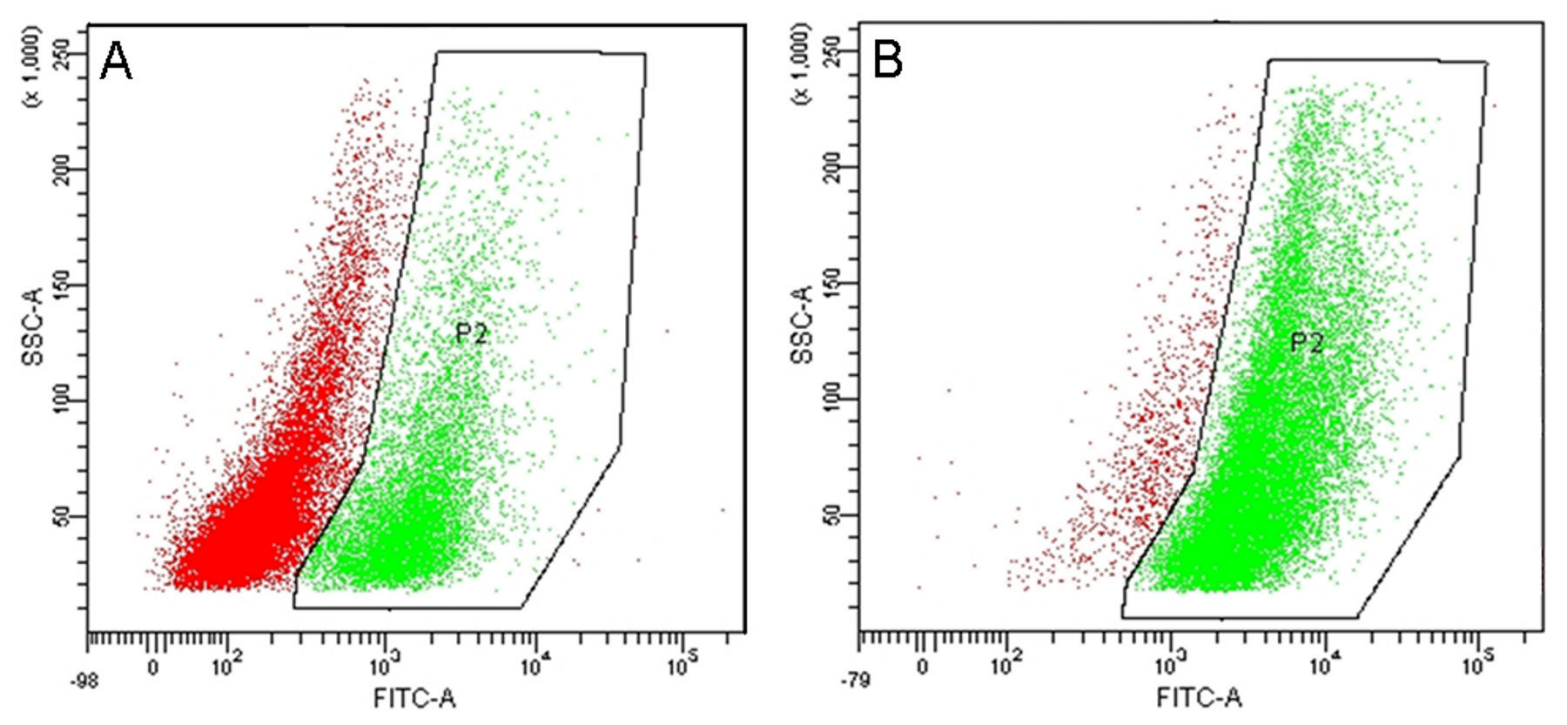
α-SMA expression in CRSwNP fibroblasts as measured by flow cytometry. Without TGF-β1 stimulation (A), with TGF-β1 stimulation (B), green marks α-SMA positive cells and red marks α-SMA negative cells.

**Figure 8:**
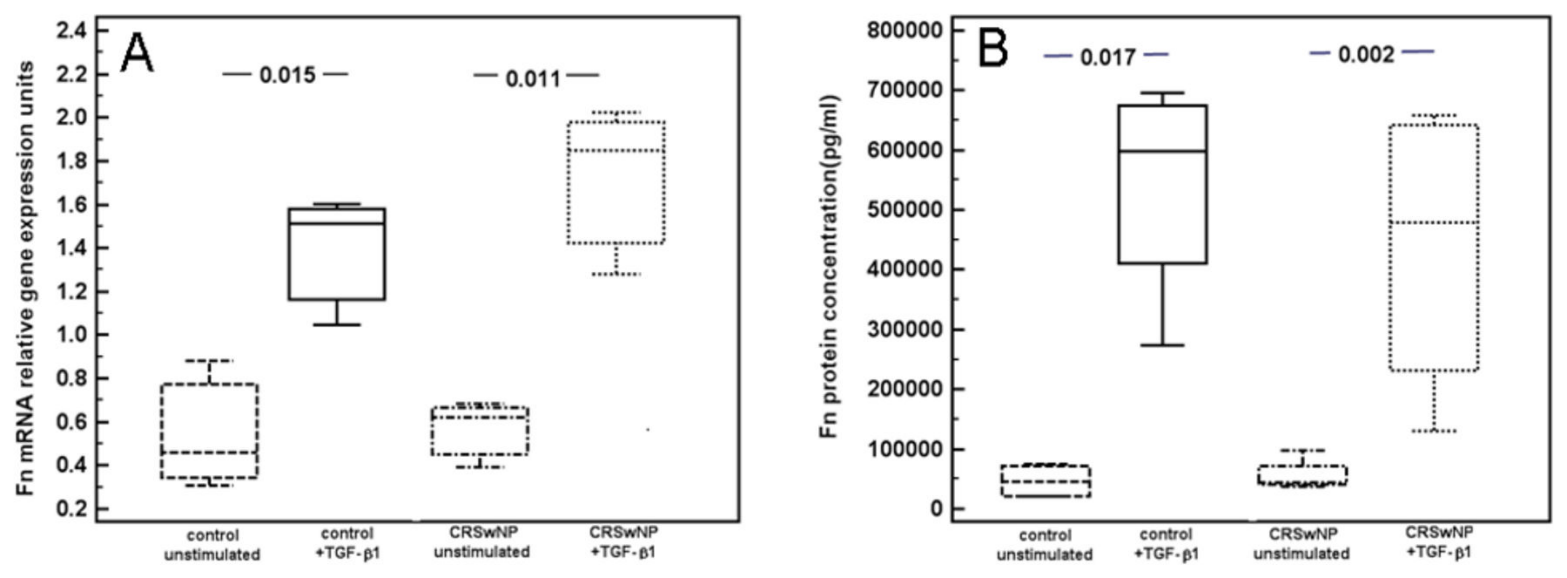
Fn mRNA expression and Fn protein concentration.

Analysis by flow cytometry demonstrated that 94.1% primary culture fibroblasts derived from CRSwNP expressed α-SMA following stimulation with TGF-β_1_, compared to 31.0% of the cells without TGF-β_1_ stimulation (Figure 7). Furthermore, TGF-β_1_ stimulation significantly increased both fibronection mRNA expression and protein concentrations in both CRSwNP and control groups, although, the levels of these markers were not significantly different between CRSwNP and control groups, before or after TGF-β_1_ stimulation (Figure 8).

## Discussion

The aim of this study was to identify the epithelial and extracellular matrix factors, which may play a role in the development and growth of CRSwNP, by comparing nasal mucosa from healthy subjects versus mature ethmoidal polyps and recently developing polyps on middle turbinate tissue of CRS patients. Our study demonstrated that compared to mature ethmoidal polyps in CRSwNP patients and normal nasal mucosa, the early stage middle turbinate polyps in CRSwNP patients suffered from significantly more severe epithelial loss . Moreover, the epithelial cell junction molecules E-cadherin, ZO-1 and occludin were expressed in significantly lower amounts in the mature ethmoidal CRSwNP group compared to healthy control mucosa group, supporting the idea that the epithelium in polyps is particularly fragile and susceptible to damage. Middle turbinate CRSwNP, however, were further characterized by the subepithelial localization of eosinophils and an increase in alternatively activated macrophages specifically in the polyp part, suggesting the presence of a macrophages enhancing stimulus in a Th2 biased inflammatory cell reaction. In contrast, an increase of TGF-β activated α-SMA expressing myofibroblasts was noted in the turbinate part of middle turbinate CRSwNP, paralleled by an increased deposition of collagen, representing a defence mechanism against inflammation and edema formation in the polyp part, particularly as there was a lack of collagen and deposition of fibronectin in the early stage polyp. To our knowledge, this is the first study that demonstrates specific cellular and extracellular factors that may play a role in the development of nasal polyps in CRS patients. 

The barrier function of the airway epithelium depends on its integrity and thus intact paracellular pathways, which is regulated by the intercellular junctional complexes consisting of tight junctions (TJs), gap junctions, adherence junctions (AJs) and desmosomes [16,17]. TJs are cellular structures located at the most apical component of the junction complex and are considered the main regulators of paracellular permeability [18]. Among the several TJ proteins, ZO-1and occludin are the most important molecules [19]. In contrast, the AJs are present in the form of a continuous belt holding neighbouring cells together by calcium-dependent cell-to-cell adhesion molecules such as E-cadherin. In accordance with the functions of these molecules, in the present study the expression of E-cadherin was found to be localized along the lateral membrane of epithelial cells, while ZO-1 and occludin were detected at the apical cell borders in linear staining patterns.

Earlier studies have indicated that events leading to polyp formation may include damage of the mucosal epithelium by mechanical forces or inflammatory mediators [20,21]. The airway epithelium is part of the innate immune system and has a prominent regulatory role in the immune response to environmental triggers [22], forming barriers against bacteria and viruses and preventing them from invading the subepithelial tissues. However, bacteria and viruses may break the epithelial barrier by decreasing the expression of junctional proteins, making it easier for the pathogens to penetrate into the tissues [23]. Using an ex-vivo human nasal mucosal culture model, Wang and colleagues [24] demonstrated that Herpes simplex virus type 1 (HSV1) infection significantly damaged the nasal epithelium and allowed the attachment of Staphylococcus aureus to the mucosa, thus facilitating the invasion of the pathogen into the nasal mucosa . Similarly, rhinovirus infections have been shown to down-regulate the transepithelial resistance of nasal epithelial cells by reducing the mRNA expression of ZO-1, E-Cadherin and occludin [25],.In addition to infection, allergen challenge can also break down TJ and AJ proteins by proteolysis [26]. Thus, in view of our findings for increased subepithelial accumulation of eosinophils and a marked increase in alternatively activated macrophages, coupled with a substantial epithelial loss in the early stage polyps, it is tempting to speculate that infectious and/or allergic agents may be involved in the initiation of polyp formation; this hypothesis awaits confirmation in future studies. 

 A markedly increased number of alternatively activated MMR-positive (M2) macrophages in the polyp area of the middle turbinate CRSwNP compared to control mucosa and mature ethmoidal CRSwNP also support the Th2 signature. Moreover, elevated expressions of CD68 [27,28] and MMR [29] in the nasal mucosa of CRSwNP has been reported in other studies. Macrophages play a key role in innate immunity by recognition, ingestion and destruction of pathogens; however, they may be polarized especially by Th cytokines and pathogens to fail in this function [30]. [31] In contrast to classically activated macrophages, alternatively activated macrophages are primed by Th2 cytokines, express the macrophage mannose receptor[32], and may support intracellular survival of bacteria and viruses [33]. Moreover, they propagate Th2 polarized immune responses and contribute to the recruitment of Th2 cells and eosinophils [34,35]. Recently, a study by Krysko and co-workers [36] found that M2 macrophages were present in increased numbers in polyps from patients with specifically Th2-biased CRSwNP compared to CRS patients without nasal polyps or control subjects. Recent studies by Takabayashi [37,38] demonstrated that coagulation factor FXIII-A was increased in NP tissue, with M2 macrophages being the major source in polyps; the authors proposed that the upregulation of FXIII-A may lead to excessive deposition of fibrin, which may run in parallel with the deposition of fibronectin. 

Here we also report an increased expression of fibronectin in mature ethmoidal polyps in CRSwNP compared to normal mucosa, and an even higher expression of fibronectin in the polyp part of middle turbinate CRSwNP. This is in accordance with the findings of other studies, which have also shown high expression of fibronectin in mature polyps in CRSwNP patients and suggested that it may contribute to edema formation and growth of nasal polyps by interaction with eosinophils [39,40]. Fibronectin plays a major role in wound healing, and cell adhesion, growth, migration and differentiation. In the lower airway fibronectin has been shown to be a chemoattractant for airway epithelial cells, regulating epithelial cell recruitment, proliferation and differentiation during epithelial repair [41,42]. As the present study has shown marked epithelial damage in middle turbinate CRSwNP, the highly increased expression of fibronectin in the polyps may reflect a repair mechanism. Furthermore, fibronectin is known to upregulate the expression of collagenase, stromelysin, and MMP9 in fibroblasts [43], as well as ECM degrading proteases involved in edema formation in CRSwNP [5,44]. In addition, it also promotes the adhesion of eosinophils via α_4_β_1_ integrin expressed on human eosinophils [45], prolonging eosinophil survival [45], and stimulates mediator release, including IL-13 and GM-CSF [6]; all activities may contribute to the localization and activation of eosinophils in polyp formation in CRSwNP patients. Moreover, fibronectin is also recognized to be the target for a large number of bacterial fibronectin-binding proteins (FnBPs) mediating the adherence of bacteria such as S. aureus to extracellular matrices as well as endothelial cells, epithelial cells and fibroblasts. Indeed, a recent study by Wang and colleagues has shown that that RV-16 infection of human nasal epithelial cells significantly increases gene and protein expression of fibronectin and augments the adherence of S. aureus [46].

Although, we did not find any marked increase in inflammation, e.g. accumulation of eosinophils and macrophages, in the turbinate part similar to that noted in the polyp part of the middle turbinate CRSwNP, we did find signs of a pronounced remodelling, as indicated by accumulation of α-SMA positive and vimentin positive myofibroblasts, as well as an increase in the number of TGF-β triggered pSmad2 positive fibroblasts. Moreover, large amount of fibres arrayed in parallel and formed thick fibre bundles in the turbinate to form a defensive wall against the polyp part.

It has been suggested that proliferation of preexisting stromal fibroblasts is the most important origin of myofibroblasts[47].When engaged in fibrogenesis, fibroblasts display the highly activated phenotype of myofibroblasts characterized by the expression of α-SMA, and resulting in the production of elevated amounts of extracellular matrix [48–51]. Central to the generation of myofibroblasts is the action of TGF-β, released locally from damaged parenchymal cells, and inflammatory cells as well as stimuli provided by matrix components such as fibronectin [48]. Also, TGF-β is a potent stimulus for the production of matrix molecules [52,53], and activation of pSmad 2 [4], a member of the Smad 2 proteins family of transcription factors demonstrated to propagate TGF-β receptor signaling [54]. Our finding of significantly more pSmad2 –positive cells being localized in the stalk part of the polyp indicates increased TGF-β signaling in this part, which can facilitate the transformation from fibroblast to myofibroblast. Indeed, our finding that stimulation with TGF-β of primary cultured fibroblasts derived from nasal polyps elevated the α-SMA expression from 31.0% to 94.1% and enhanced fibronectin production, supports the role of TGF-β in the enhancement of myofibroblast differentiation and ECM production , possibly forming a fibrotic wall as a defensive mechanism to prevent edema and inflammation from spreading. 

 Chronic airway remodelling has been suggested to be the result of an aberrant repair cycle which occurs in response to chronic airway inflammation. However, evidence from studies over the last decade suggests that inflammation and remodelling occur in concert, rather than remodelling being a consequence of long-standing airway inflammation [55]. Airway biopsy studies have shown that reticular basement membrane thickening is already present in young children with difficult asthma without any associations with age, symptom duration or eosinophilic airway inflammation [56]. Our group has recently compared remodelling and inflammatory patterns in Caucasian and Chinese CRSwNP patients and demonstrated that there were clear differences in the inflammatory patterns in these ethnicity groups [7] while the remodelling patterns were similar, indicating a dissociation between inflammation and remodelling [5]. From this study, we may also conclude that remodelling occured in parallel, rather than subsequent to inflammation.

This study is somewhat limited due to the comparatively small numbers of samples with polyp formation on the medial aspect of the middle turbinate in patients with bilateral CRSwNPs, as this condition is not frequent to find. These polyps are developing late in the disease process, but were formed only recently before the surgery; they are of younger age (early stage) than the mature ethmoidal specimens from CRSwNP patients. Furthermore, biopsies of the middle turbinate CRSwNP were limited in size, which restricted other possible investigations. However, despite these limitations, we were able to demonstrate significant differences in the inflammatory cells and ECM deposition between early and late stage nasal polyp disease, suggesting that this merits confirmation in further investigations involving larger patient cohorts recruited from several study centres across the country.

## Conclusion

 In summary, our study has indicated a number of pathophysiologic differences between the early stage polyps and mature polyps that have not been described before in CRSwNP patients. The epithelial loss was more prominent in the early stage polyps in the middle turbinate in CRSwNP patients, coupled with increased numbers of especially M2 type macrophages and markedly high expression of Fibronectin. Taken together, these findings suggest that aggravated epithelial damage and Fibronectin expression play a crucial role in the adhesion and penetration of the basement membrane by bacteria in the initial stages of polyp formation. Similarly, the increased numbers of macrophages in the polyp area of the middle turbinate CRSwNP suggests a defective host defense mechanism in the early stage of the disease. In contrast, a fibrotic response build up a defence mechanism involving increased deposition of dense collagen fibre bundles in the underlying mucosa to prevent spread and generalization of edema and inflammation. Overall, these findings suggest a complex network of processes in the formation of the CRSwNP; including gross epithelial damage and repair reactions, eosinophil and macrophage cell infiltration, and tissue remodelling. Furthermore, remodelling appears to occur in parallel, rather than subsequent to inflammation, as has been shown in CRS patients without nasal polyps [57]. 
